# Impacts of Biogas Technology Adoption on Rural Household Energy Expenditure in South Ethiopia

**DOI:** 10.1155/tswj/2846569

**Published:** 2025-02-15

**Authors:** Lemma Shallo, Getachew Sime

**Affiliations:** ^1^Department of Economics, Wolkite University, Wolkite, Ethiopia; ^2^Department of Biology, Hawassa University, Hawassa, Ethiopia

**Keywords:** biogas technology, domestic energy, energy expenditure, household income

## Abstract

Despite the fact that there is a large number of biogas plants built in Ethiopia, their contribution in reducing energy expenditure and increasing income has not been fully examined. Therefore, this study examined the impacts of biogas technology adoption on rural household energy expenditure in South Ethiopia. Data were collected from 246 sample households, 123 biogas adopters, and 123 nonadopters. Simple random and purposive sampling techniques were used to select sample households. For data analysis, propensity score matching and binary logistic regression models were used. The logistic regression results showed that access to credit (*p* ≤ 0.01), access to electronic media (*p* ≤ 0.05), and farm size influenced the adoption of biogas technology positively and significantly (*p* ≤ 0.05), whereas distance to water sources influenced negatively and significantly (*p* ≤ 0.05). The average treatment of the treated unveil that biogas technology adoption reduced household annual energy expenditure, and enhanced household annual income by reducing money spent on fuel purchases. Therefore, the deployment of biogas technology improves rural households' energy supply and increases their annual income. To promote biogas technology adoption and increase rural energy security and income, government energy offices, policymakers, and development partners should capitalize on variables influencing biogas technology uptake.

## 1. Introduction

In 2021, fossil fuels supplied over 80% of the global energy demand. Historically, the world has relied on oil, natural gas, and coal to supply its energy needs, with oil accounting for 30.95%, natural gas accounting for 24.42%, and coal accounting for 26.90% [1].

Global and regional trends imply that renewable energy sources will soon be widely deployed to meet energy demand [2]. Renewable technologies are clean energy sources whose optimal use is sustainable in terms of present and future economic, social, and societal needs [3]. Therefore, appropriate policy implementation, enabling technologies, and investments in renewable energy resources have the potential to provide benefits such as reduced greenhouse gas emissions, sustainable electricity generation, and the provision of an economic justification for various stakeholders to invest in the use and promotion of renewable energy technologies [4].

An important and promising strategy to address the energy and environmental challenges is the production of bioenergy from agricultural wastes. This process involves converting various types of agricultural residues and by-products into bioenergy, which can be used as renewable and cleaner alternatives to nonrenewable energy [5, 6]. Moreover, the combustion process of biomass is of great importance as the world moves away from the use of fossil fuels to encompassing renewable energy that is environmentally friendly [6, 7]. The improvement in clean energy and the long-term progress in the energy sector hinge on the crucial processes of generating, transporting, and storing energy [8]. Therefore, these transformative technologies in clean energy generation from biomass wastes, distribution, and storage provide valuable insights for sustainable energy production and utilization system [5–7].

Around 2.8 billion people worldwide use solid fuels for cooking [9]. In Ethiopia, around 81.4% of households cook with wood, 11.5% with leaves and dung cakes, and 2.4% with kerosene [10]. This demonstrates that traditional biomass is the primary source of energy in Ethiopia. This is one of the reasons why boosting access to modern energy services has been a significant component of sustainable development efforts in developing countries. One of the desirable and feasible choices has been the promotion of renewable energy. This is due in part to the fact that via the application of renewable energy technology, sustainable renewable energy may be generated from locally available and affordable organic resources [11]. In this sense, a biogas plant is one of the renewable energy technologies, which has been suggested to provide reliable supply of contemporary electricity to rural populations in sub-Saharan Africa [12].

Ethiopia has a considerable number of biogas facilities under construction. However, basic issues regarding how far biogas plant installations have contributed to rural household welfare by lowering energy expenditure and raising income have not been studied adequately. A few research studies have been conducted on biogas energy in Ethiopia, which include among others, feasibility study of the national domestic biogas [13]; a cost-benefit analysis of biogas energy in Sidama Region and Oromia region [14]; the diverse environmental impacts of domestic biogas technology in North Ethiopia [15]; the contribution of producing and using biogas to mitigating GHG emissions [10]; the effects of switching from dung combustion to a biogas system using the life cycle assessment approach on the environment [16]; factors determining the functional status of family size biodigesters in South Ethiopia [17]; and adoption of biogas technology and the state of household health in North Ethiopia [18]. Despite this, none of the research paid sufficient attention to the impact of the deployment of biogas technology on rural household energy expenditure and associated conditions. As a result, the objective of this study was to explore the factors that influence biogas technology adoption and the effects on household energy expenditure in rural communities in South Ethiopia.

## 2. Methodology

### 2.1. Study Area Description

**Figure 1:**
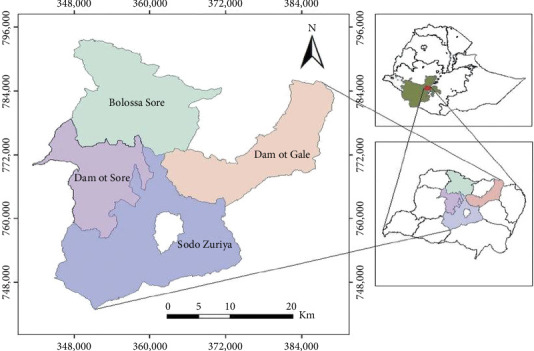
Physical map of the study site.

The study was undertaken in South Ethiopia (Figure 1), in Wolaita Zone, in ten kebeles1 of the Sodo Zuria district. The district is one of the 12 administrative districts of Wolaita Zone with almost similar agroecology and demography. Out of 186,779 total population of the district, 49.17% are male and the remaining 50.83% are female, and the total average household size is 6 individuals [19]. Among these households, only 0.1% of them adopted biogas technology since 2011 [20].

The district covers 41,927 ha among which 53.26% is used for annual crop production, 12.95% is used for perennial crop production, 4.43% is covered by forest, and remaining 6.47% is used for other purposes. Livestock keeping, which includes cattle, sheep, goats, horses, donkeys, mules, and poultry, is a highly widespread practice. About 9440 (22.53%) ha land is used for grazing. Agriculture is the mainstay of livelihood in the district. The district experiences midland (65%) and highland (35%) agroecology, which is suitable for diverse agricultural production. Therefore, mixed farming of crop production, livestock keeping, and beekeeping are the major sources of income and livelihood of the rural communities [21].

### 2.2. Methodologies

**Figure 2:**
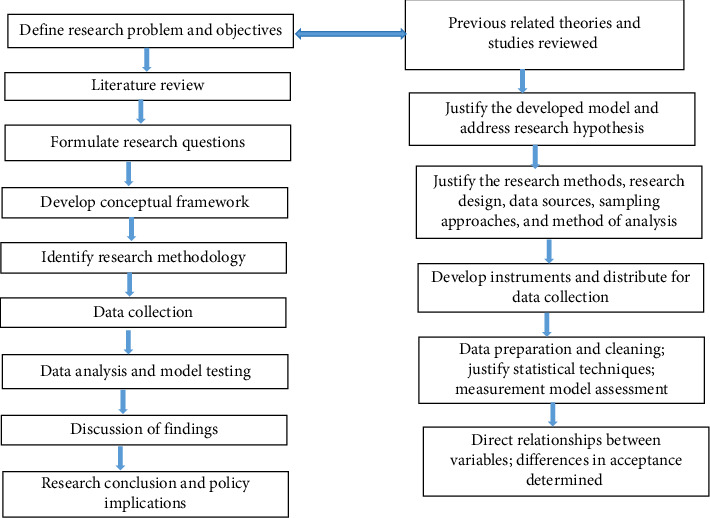
Research flow chart.

The research flowchart (Figure 2) displays the process carried out to accomplish the study intended. It is a diagrammatic sketch exhibiting the flow of the activities executed.

#### 2.2.1. Source of Data and Method of Collection

Data were collected from both primary and secondary sources. Interview questionnaires were used to acquire primary data from the sample households. Secondary data were also gathered from published sources, including journal articles.

#### 2.2.2. Sampling Technique and Sample Size Determination

This study's unit of analysis was both the biogas adopter and nonadopter sample households. Simple random and purposive sampling techniques were employed to identify sample households. The simple random sample technique of the lottery approach was used to pick biogas adopter households. The nonadopter samples were chosen using the purposive sampling technique [22].

Following the Ethiopian national recommendation for biogas adoption, only households with four or more livestock and with access to appropriate water supply (30–40 min) were considered as biogas adopters [23]. Moreover, each sample biogas adopter household had one nearest neighbor, which was purposively selected as a nonadopter household. Accordingly, 123 sample households from each category were chosen, totaling 246 sampling households for the study. The main justifications behind using purposive sampling technique in selecting the sample nonadopter households in the study area is the relative sizes of biogas adopter households, which is quite disproportional to the size of potential biogas adopter households [20]. Hence, purposive sampling technique assisted to avoid the occurrence of highly disproportionate sample size of biogas adopter and nonadopter households in the district. Purposive sampling also helped to exclude households from the remotest rural villages where the biogas program had never reached yet.

There were a total of 180 households who already adopted the biogas technology in the study district [21]. Therefore, among them, 123 adopter sample households were chosen. The sample size for biogas adopter households was determined using the general method developed by the Air University (AU) [24]. It was determined with a 95% confidence and a 5% precision level:

(1) $$\begin{array}{c} n=\frac{{NZ}^{2}*0.25}{d^{2}\left(N-1\right)+\left(Z^{2}*0.25\right)}=\frac{180{\left(1.96\right)}^{2}*0.25}{{\left(0.05\right)}^{2}*179+{\left(1.96\right)}^{2}*0.25}=\frac{172.872}{1.4079}=123, \end{array}$$

where *Z* is the number of standard deviation units of the sampling distribution that corresponds to the desired precision level (1.96), *n* is the sample size, *N* is the size of the total population, and *d* is the accuracy level (0.05).

#### 2.2.3. Data Collection

The primary data were collected through administering a pretested semistructured questionnaire to the 246 sample households.

#### 2.2.4. Method of Data Analysis and Estimation Approach

Depending on the objectives of the study, descriptive and inferential statistical methods were used to analyze the data. The descriptive statistics used were percentages, means, variances, standard deviations, frequency distributions, and graphs, whereas the inferential statistics used a logistic regression model and propensity score matching (PSM) approach for data analysis. The institutional, social, and economic characteristic features between the adopters and nonadopters were compared using *t*-test and *χ*^2^-test for continuous and discrete variables, respectively.

##### 2.2.4.1. Binary Logistic Regression (BLR)

This model was used to assess the main factors impacting household decisions on biogas technology adoption. Logistic regression is a probability estimation method used when the dependent variable is binary and the independent variable is at any size [25]. If Y is the dependent variable, it can assume a value of either 1 or 0.

(2) $$\begin{array}{c} \text{Bi}\left\{\begin{array}{cc} =1, & \text{if }a\text{ household }i\;o\text{wn}s\text{ bioga}s\text{ plant},\\ =0, & \text{otherwise}. \end{array}\right. \end{array}$$

Therefore, the following is the logistic regression model for calculating the probability of adopting biogas technology (Pi):

(3) $$\begin{array}{c} \mathrm{Pr}\left(Bi=1\right)=P_{i}=\frac{1}{1+\left(e^{zt}\right)}. \end{array}$$

The likelihood of households of not adopting biogas technology

(4) $$\begin{array}{c} \mathrm{Pr}\left(\text{Bi}=0\right)=1-P_{i}=1-E, \end{array}$$

when dividing (3) by (4), it gives odds ratio: (*Pi*/1 − *Pi*).

The logit model uses an odds ratio that has been transformed logarithmically:

(5) $$\begin{array}{c} \text{Li}=\text{nl}\left(\frac{PI}{1-Pi}\right)=Z_{i}=\alpha +\beta 1X1+\beta 2X2+\cdots +\beta kXk+\varepsilon i, \end{array}$$

where *X*1, *X*2,…, *Xk* are explanatory variables which influence the adoption of biogas technology; 1, 2,…, *k* are estimated parameters corresponding to each explanatory variable; *k* is the number of explanatory variables; and *i* is the random error.

##### 2.2.4.2. PSM Analysis

Impact assessment of a designed program intervention is to show the effect of the program on participant group and comparator group that does not participate in the program as a control group, with similar preintervention socioeconomic characteristics. In order to evaluate the impact of a particular program, its effect is separated from any other elements that may have an impact on the outcome [26].

The PSM method was used to evaluate the impact of participation in the biogas program in the research area because there was no such design in this study. The PSM approach has become the most extensively used nonexperimental instrument for assessing the effectiveness of social and economic programs [27]. It is based on identifying a group of treated individuals (participants) who share all relevant pretreatment characteristics with the control group (nonparticipants). The important distinction is then participation versus nonparticipation in the biogas program.

The potential outcome method or the Rubin model is used as a basic framework for evaluating and formalizing this topic [27]. Individuals, therapy, and prospective results are the model's cornerstones. In the event of a binary treatment, the treatment indicator Di equals 1 if the individual *i* receives therapy (participates in the biogas program) or 0 otherwise.

Then, for each person, the potential outcomes are specified as Bi (Di), where *i* = 1,…, *N*, and *N* is the total sample size. Ti is characterized as the difference between a person's potential outcomes with treatment versus that person's potential outcome without treatment:

(6) $$\begin{array}{c} T_{i}=B_{i}\left(1\right)-B_{i}. \end{array}$$

The average treatment effect on the treated (ATT), which assesses the effect of the biogas initiative on the energy consumption of those participating households, is the main measurement of interest:

(7) $$\begin{array}{c} \text{ATT}=E\left(\frac{T}{D}=1\right)=E\left[B\left(1\right)\left|D=1\right.\right]-E\left[B\left(0\right)\left|D=1\right.\right], \end{array}$$

where *E* represents the average (or expected value).

The propensity score (PS) = P[D = 1/X] = P[X], which is the likelihood that a person will take part in a treatment given the observation covariates *X*, is an alternative balancing score. One way to express conditional independence assumption (CIA) is by

(8) $$\begin{array}{c} \left(\text{Un}-\text{confoundedness given PS}\right)\left[B\left(0\right),B\left(1\right)\frac{\Pi D}{p\left(X\right)}\right]\text{AX}. \end{array}$$

The common support area is defined as the area with the lowest and highest PSM for the treatment and control group households, respectively. Any observations where PSM is less than or higher than the minimum and maximum values for the treatment and control, respectively, must be omitted [28].

Match pairs were generated utilizing multiple ways of matching estimators based on estimated probability of participation. The impact is then calculated using the difference between the simple means of the relevant outcome variable for participant and nonparticipant households. Consequently, the impact of the biogas scheme on household energy consumption was investigated. Identifying a match for a participant household, based on a vector of characteristics, is similar to finding a match based on the probability of participation in the biogas program and on the vector of household characteristics, i.e., P(X_i) = Pr(Bi = 1/X_i). As a result, the average impact of biogas technology adoption on household energy expenditure is given by

(9) $$\begin{array}{c} \Delta_{i}=\frac{\sum_{j=1}^{p}Y_{\text{ij}1}-\sum_{i=1}^{\text{NP}}Y_{\text{ij}0\text{⁣}}}{P}, \end{array}$$

where *P* denotes the total number of participants and NP denotes the total number of nonparticipants, Y_j1_ denotes the total annual energy expenditure of participant household *j*, and Y_ij0⁣_ denotes the total annual energy expenditure of the *i*th nonparticipant household that was matched to the *j*th participant households.

#### 2.2.5. Definition of Variables and Hypothesis

##### 2.2.5.1. Treatment Variables

If a household has biogas plant, the binary variable has a value of 1; otherwise, it has a value of 0. The conditional probability of participation was calculated with the help of the logit model.

##### 2.2.5.2. Outcome Variables

It is a continuous variable, representing household annual energy expenditure (expense for fuel). It is the actual value of energy sources consumed. It was hypothesized that the energy expenditure for participants of biogas program would be less than the counterparts (nonadopters). The impact (outcome) was evaluated using the PSM model.

##### 2.2.5.3. Explanatory Variables

Table 1 presents the description of demographic, socioeconomic, biophysical, and institutional variables which are presumed to determine biogas technology adoption and household energy expenditure and their assumed relationship.

## 3. Results and Discussion

### 3.1. Descriptive Statistics Results

The adoptions of biogas technology and energy consumption are predicted to be influenced by the demographic and socioeconomic traits of the sample households.

#### 3.1.1. Household Head Age

The age of sample households ranged between 28 and 65 for adopters and 35 and 60 for nonadopters. The age difference between household heads who adopted biogas in the sample and those who did not was statistically significant (Table 2). This demonstrates that age of household head can encourage or discourage biogas technology adoption. This result agrees with findings from previous studies that age of household head significantly and positively influenced biogas technology adoption [15] and positively influenced biogas technology adoption [29, 30].

#### 3.1.2. Education Level

Illiterate (those who cannot read or write) and literate sample household heads made up 10.4% and 89.4% of the total sample household heads, respectively. The sample household heads' educational level in years of schooling ranged from 0 to 13, with an average of 5.5 years. In particular, the sample biogas adopter and nonadopter household heads had a mean educational level of 5.8 and 5.2 years of schooling, respectively. The mean educational level difference between the adopter and nonadopter household heads was statistically negligible (Table 2). This means that the education level of household heads has no effect on the adoption of biogas technology. Several prior researches, however, found a significant and positive association between household heads' education level and biogas technology adoption [31]. According to the study's findings, access to information, such as access to electronic media and new technology, has a greater influence than the education level of household heads.

#### 3.1.3. Household Size

The sample household size ranged from 5 to 9, with an average of 6 people. The adopter and nonadopter households each had an average household size of roughly 6 people. The average household size difference between adopter and nonadopter households was statistically negligible (Table 2). This finding implies that household size, whether large or small, has no effect on the adoption of biogas technology. This outcome is consistent with earlier research [32]. It does, however, contradict prior findings [29, 30]. This could be because the study site is in one of Ethiopia's most heavily inhabited districts. The family size between both categories does not show a difference and hence does not influence the decision to adopt a technology. In such a situation, it is rather other factors, such as farm size, income, access to credit facilities, livestock size, and so on, that influence their decision to adopt biogas technology.

#### 3.1.4. Cattle Size in TLU

The sample households in TLU had an average herd size of 3.1. The adopter and nonadopter households' average cattle size in TLU was 3.6 and 2.6, respectively. The mean difference in cattle size between adopter and nonadopter households was statistically significant (Table 2). This result demonstrates that cattle size influences biogas technology adoption. That is, households keeping more cattle size are more encouraged to adopt biogas technology compared to their counterparts. This is in agreement with several findings from previous studies [20, 32].

#### 3.1.5. Total Household Income

During the study period, the average annual income of the sample households was 23,854.60 ETB. The average annual income of adopter and nonadopter households was 29,692.30 ETB and 18,016.90 ETB, respectively. The difference in average income between adopters and nonadopters was statistically significant (Table 2). This result displays that annual income influences biogas technology adoption, which in turn implies that households having higher income are more likely to adopt biogas technology [33, 34].

#### 3.1.6. Farmland Size

The sample households' average farmland size was 0.8 ha. The adopter and nonadopter households' average cropland size was 1 ha and 0.6 ha, respectively. The average farmland size difference between adopter and nonadopter households was statistically significant (Table 2). This demonstrates that the size of a farmland affects a household's decision to adopt biogas technology. This demonstrates that households with more farmland have enough backyard space to put biodigesters and are more likely to adopt biogas technology. This result is in agreement with findings from previous studies [18, 35].

#### 3.1.7. Sex of Household Head

Households headed by females constituted 13.4% of the total sample households and only 13.8% of sample biogas adopters.  Table 3 shows that the mean difference in headship is statistically negligible. This implies that the gender of household heads has no influence on the decision to employ biogas technology, which contradicts prior research findings [30, 36].

#### 3.1.8. Access to Credit Service

Credit was available to 79.3% of the total sample households. About 92.7% of the adopter and 65.9% of the nonadopter households had access to credit service, which is statistically significant (Table 3). This demonstrates that availability of finance facilities influences the decision to adopt biogas technology. This means that having access to finance services encourages households to embrace biogas technology. This is consistent with several findings from previous studies [37, 38].

#### 3.1.9. Access to Electronic Media

Nearly 86.2% sample adopter and 57.7% nonadopter household heads had access to electronic media, which is statistically significant (Table 3). This indicates that household heads that have access to electronic media are more likely to get adequate information and to adopt biogas technology. This finding is consistent with past research findings [30].

### 3.2. Econometric Regression Analysis

#### 3.2.1. Logit Model Estimation Results and Discussion

Table 4 displays the BLR results for biogas technology adoption factors.

#### 3.2.2. Access to Credit

According to the logistic regression, access to financing influenced biogas technology adoption positively and significantly (*p* ≤ 0.1) (Table 4). Keeping all other variables equal, access to finance by households enhanced the likelihood of biogas technology adoption by a factor of 2.34. This demonstrates that access to financing improves household affordability in adopting biogas technology. Previous research backs up this finding [15, 18, 39]. This validates that the provision of credit services in rural regions is likely to alleviate financial barriers to implementing biogas technology and managing biodigesters.

#### 3.2.3. Farm Size

Farm size had a significant (*p* ≤ 0.05) positive impact on biogas technology uptake (Table 4). That is, as household farm size increased by 1 ha, the likelihood of adopting biogas technology increased by a factor of 2.60. These data indicate that farm size is one factor determining biogas technology adoption. This finding is consistent with the findings that farm size promotes the adoption of biogas technology in a positive way [35, 40].

#### 3.2.4. Distance to Water Sources

Distance to water source and biogas technology adoption had a negative significant (*p* ≤ 0.01) correlation (Table 4). As the distance to water source from home increased by 1 minute, the likelihood of biogas technology adoption decreased by a factor of 0.91, with other factors being constant. This means that the distance from a water supply is an important factor in determining biogas technology adoption. This finding is consistent with prior research that found a negative significant association between distance to the nearest water source and biogas technology adoption [18, 41].

#### 3.2.5. Access to Electronic Media

The estimation result demonstrated a positive significant (*p* ≤ 0.05) association between access to electronic media and biogas technology adoption, as hypothesized. Keeping all other parameters constant, access to electronic media enhances the likelihood of biogas technology adoption by a ratio of 2.46 when compared to the counterparts. In this regard, there are national and regional radio programs that broadcast information on the use of biogas technology. This finding is consistent with earlier research [30, 42]. Technology adoption is stronger in households who have access to knowledge and awareness via various communication channels than their counterparts.

#### 3.2.6. Estimation of Propensity Scores

For the anticipated matching exercise, the estimated model appears to perform well (Table 5). The pseudo-R^2^ value was discovered to be 0.3267. If participant households have few differentiating characteristics, as indicated by a low pseudo-R^2^ score, it will be simpler to match treated and untreated households.

#### 3.2.7. Matching Participants and Nonparticipants

The pseudo-*R*^2^ measures how well the regressors explain the likelihood of adoption (Table 6). There was no consistent variation in the distribution of variables between the two groups after matching. As a result, the pseudo-*R*^2^ was relatively low [28].

Table 6 shows the common support region, which ranged from 0.0133847 to 0.8967754. In other words, families with estimated propensity scores less than 0.0133847 but greater than 0.8967754 were excluded from the matching process. This restriction resulted in the elimination of 5 treated and 3 untreated households. This demonstrates that the study did not exclude many households from the sample when calculating the effect estimation.

**Figure 3:**
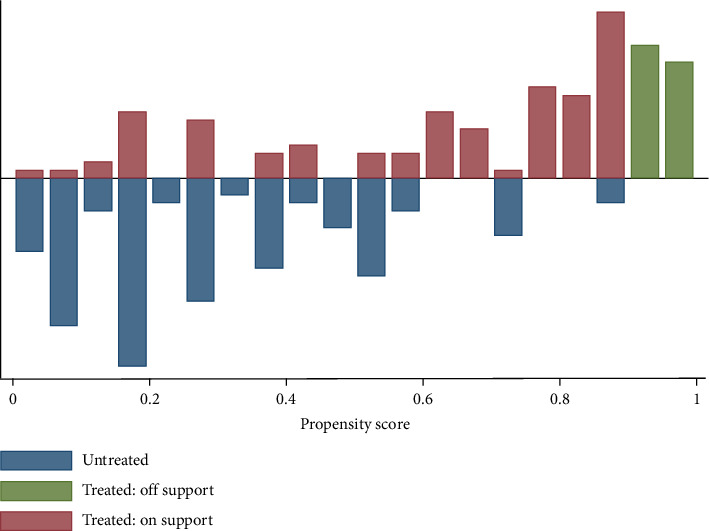
Propensity score distribution and common support region. *Source:* Author's own (2018). *Note:* Figure depicts the common support region where the propensity scores (between 0 and 1) of treated group (above the line with red bars) match with the untreated one (below the line with dark blue bars). Those propensity scores which do not match will be out of the common support region (the green bars). Moreover, the tallness and shortness of the bars shows the intensities of samples' propensity scores.

The findings in the nonadoption and adoption of biogas technology groups that have a suitable comparison are indicated in blue (untreated on support) and red (treated on support), respectively, whereas the green color (treated off support) denotes observations in biogas technology uptake participants (Figure 3).

#### 3.2.8. Choice of Matching Algorithm

In terms of the above quality indicators, caliper 0.01 matching produced a comparatively low pseudo-R^2^ with the best balancing test and a large matched sample size when compared to other alternative matching estimators. This was chosen as the best matching estimator (Table 7).

#### 3.2.9. Testing the Balance of Propensity Score and Covariates

In virtually all cases, it is clear that sample differences in unmatched (before matching) data outnumber those in matched case samples. As a result of the matching process, the nonparticipant and participant sample houses had a high degree of covariate balance that is ready to be used in the estimation methods. Similarly, t-values (Table 8) reveal that before matching, nearly half of the selected variables demonstrated statistically significant differences, whereas after matching, virtually all of the covariates were balanced and statistically insignificant (Table 8).

All of the aforementioned tests indicate that the chosen matching method best fits the data, which aided in estimating ATT for the treated for the sample households of biogas adopters and nonadopters.

#### 3.2.10. Estimating ATT: Household Energy Expenditure Impact Estimate of Biogas Technology Adoption

The estimation reveals that biogas adoption had a considerable positive influence on the well-being of biogas adopting families by lowering household energy expenditure. According to the ATT, adopting households lowered their annual energy expenditure by 461 ETB per year on average when compared to nonadopter households (Table 9). This contributes to the primary goal of implementing biogas technology, which is to reduce fuel expenses, and raises household annual income by saving energy costs [13, 23]. Adoption of biogas technology minimizes household energy expense [18, 30, 33, 43].

Adopter households spent much less on energy than nonadopter households (Table 9). Furthermore, cutting energy expenditure significantly raised adopter households' income when compared to nonadopter households.

## 4. Conclusion and Policy Recommendations

The primary objective of this study was to assess the factors influencing the adoption of biogas technology and its impact on energy consumption in rural households. According to the logistic regression results, access to credit, market, electronic media, and distance to water sources all had a substantial impact on biogas technology adoption. Except for the distance to water sources, the remaining criteria influenced adoption positively. This demonstrates that the influence of biogas technology is closely tied to factors such as credit service availability, farm size, distance to water sources, and access to electronic media. Furthermore, the average treatment of the treated found that biogas technology adopting families cut their yearly energy expenditure by 461 Ethiopian Birr per year on average when compared to nonadopting households. Adoption of biogas technology cuts family fuel prices and raises annual income by saving money on fuel purchases. As a result, the employment of biogas technology helps significantly to the development of household welfare by lowering energy costs and increasing income. In addition to enabling household self-sufficiency in the generation and utilization of energy, adopting families gain the following additional advantages from the use of biogas technology: Fast cooking saves time for other household chores (like collecting firewood), reduces deforestation for firewood, provides high-quality fertilizer, reduces greenhouse gas emissions from agriculture and forests, mitigates climate change, improves women's health because there is less indoor air pollution, increases agricultural production, and ultimately improves livelihoods. The simultaneous delivery of these advantages makes biogas technology special. However, governments, nongovernment organizations, international organizations, and local communities do not give biogas technology the level of attention it deserves in terms of research and development or the advantages it may provide. Additionally, its main products, biogas and bioslurry, have received a disproportionate quantity of attention while they are equally important. This is because the well-being of millions of inhabitants in the developing world is currently severely hampered by both access to electrical energy and the cost of chemical fertilizers.

## Figures and Tables

**Table 1:** Summary of explanatory variables and their presumed signs.

Variable	Type	Description	Expected sign
Age (AGE_HH⁣^∗^)	Continuous	Age of the household head	±
Sex (SEX_HH)	Categorical	Sex of the household head: female = 1; male = 0	±
Education (EDU_HH)	Continuous	Household head's educational level in years of schooling	+
Household size (HH_SIZE)	Continuous	Total number of people in the household	±
Livestock ownership (CATT_SIZE)	Continuous	Livestock owned by HH in TLU⁣^∗∗^	+
Total income (INCOME_TOT)	Continuous	Total annual income of the household	+
Access to credit (CREDIT_ACC)	Categorical	Having access to credit = 1; otherwise = 0	+
Farmland size (FARM_SIZE)	Continuous	Household's total farmland owned in hectare	+
Distance to water sources (DIST_WAT)	Continuous	Walking distance of the main water source from home in minutes	−
Access to electronic media (ELE_MEDIA)	Categorical	Have radio and/or television = 1; otherwise = 0	+

⁣^∗^HH stands for household head.

⁣^∗∗^TLU stands for tropical livestock unit, with the values of ox = 1, cow = 1, horse = 1.1, heifer = 0.75, calf = 0.25, donkey = 0.7, sheep = 0.13, goat = 0.13, mule = 0.7, and poultry = 0.013.

**Table 2:** Summary statistics for continuous variables of the household socioeconomic characteristics.

Dependent variable: biogas energy adoption (*B*_*i*_ = 1, adopter; *B*_*i*_ = 0, nonadopter)							
Independent variables	Total sample households		Adopters		Nonadopters		*T*-test
	Mean	SD	Mean	SD	Mean	SD	
Age of HH head	45.2	7.6	43.9	7.5	46.4	7.6	2.60⁣^∗∗∗^
Education level	5.5	3.2	5.8	2.9	5.2	3.5	−1.48⁣^∗∗^
Household size	5.6	1.6	5.7	1.5	5.5	1.7	−0.87⁣^∗^
Cattle size	3.1	1.4	3.6	1.5	2.6	1.2	−5.65⁣^∗∗∗^
Total HH income	23,854.6	17,175.6	29,692.3	20,305.1	18,016.9	10,554.9	−5.66⁣^∗∗∗^
Farm size (ha)	0.8	0.6	1.0	0.8	0.6	0.3	−4.89⁣^∗∗∗^
Distance to water source	21.1	11.0	16.8	10.1	25.4	10.2	6.60⁣^∗∗∗^

*Note:* Source: author's own (2018).

⁣^∗∗∗^, ⁣^∗∗^, and ⁣^∗^ represent significant at 1%, 5%, and 10% significance level, respectively.

**Table 3:** Mean comparison for discrete variables.

Dependent variable: biogas energy adoption (*B*_*i*_ = 1, adopter; *B*_*i*_ = 0, nonadopter)						
Independent variable		Adopter		Nonadopter		X^2^-value
		Frequency	Percent	Frequency	Percent	
Sex of HH head	Female	17	13.8	16	13.0	0.04
	Male	106	86.2	107	87.0	

Access to credit	Yes	114	92.7	81	65.9	26.94⁣^∗∗∗^
	No	9	7.3	42	34.1	

Access to electronic media	Yes	106	86.2	71	58.2	23.92⁣^∗∗∗^
	No	17	13.8	51	41.8	

*Note:* Source: author's own (2018).

⁣^∗∗∗^Significant at 1% significance level.

**Table 4:** The binary logistic regression results for biogas technology adoption factors (Bi).

Bi	Coef.	Std. err.	*z*	*P* > *z*	Odds ratio
AGE_HH	−0.0446632	0.0289033	−1.55	0.122	0.9563195
SEX_HH	0.2175151	0.5610355	0.39	0.698	1.242984
EDU_HH	0.0207061	0.0663884	0.31	0.755	1.020922
HH_SIZE	0.0321022	0.1030279	0.31	0.755	1.032623
CATT_SIZE	0.1419412	0.1632736	0.87	0.385	1.152509
INCOME_TOT	0.001	0.001	1.12	0.262	1.000017
CREDIT_ACC	0.85029	0.4988012	1.70	0.088⁣^∗^	2.340325
FARM_SIZE	0.9545448	0.4687577	2.04	0.042⁣^∗∗^	2.597488
DIST_WAT	−0.0867462	0.0194825	−4.45	0.001⁣^∗∗∗^	0.9169097
ELE_MEDIA	0.901866	0.4405687	2.05	0.041⁣^∗∗^	2.464197
_Cons	0.4754569	1.407909	0.34	0.736	1.608749
Number of obs. = 245		LR chi2 (10) = 102.56		Prob. > chi2 = 0.001	
Log likelihood = −118.53943		Pseudo *R*^2^ = 0.3020			

*Note:* Source: own computation (2018).

⁣^∗∗∗^, ⁣^∗∗^, and ⁣^∗^ represent significant at 1%, 5%, and 10% significance level, respectively.

**Table 5:** Propensity score matching estimation using logistic regression.

Bi	Coef.	Std. err.	*z*	*P* > *z*
AGE_HH	−0.0391898	0.0296297	−1.32	0.186
SEX_HH	0.1790833	0.5889356	0.30	0.761
EDU_HH	0.011717	0.066981	0.17	0.861
HH_SIZE	0.0415674	0.1058812	0.39	0.695
CATT_SIZE	0.1529276	0.1683247	0.91	0.364
INCOME_TOT	0.001	0.001	0.84	0.402
CREDIT_ACC	0.9439375	0.5026938	1.88	0.060⁣^∗^
FARM_SIZE	0.7628916	0.4667195	1.63	0.102
DIST_WAT	−0.0849677	0.0198258	−4.29	0.001⁣^∗∗∗^
ELE_MEDIA	0.9444597	0.4519496	2.09	0.037⁣^∗∗^
MARK_ACC	1.119745	0.3624598	3.09	0.002⁣^∗∗∗^
_Cons	−0.0922253	1.459903	−0.06	0.950
Number of obs. = 238		LR chi2(11) = 107.80		Prob. > chi2 = 0.001
Log likelihood = −111.05987		Pseudo *R*^2^ = 0.3267		

*Note:* Source: own computation (2018).

⁣^∗∗∗^, ⁣^∗∗^, and ⁣^∗^ represent significant at 1%, 5%, and 10% significance level, respectively.

**Table 6:** Distribution of estimated propensity scores of sample households.

Group	Observation	Mean	STD	Minimum	Maximum
All households	238	0.4957983	0.3135935	0.0133847	0.9863986
Biogas adopters (treated)	118	0.6954069	0.2679581	0.0133847	0.9863986
Nonadopters (control)	120	0.2995166	0.2165677	0.0133847	0.8967754

*Note:* Source: author's own result (2018).

**Table 7:** Performance measures of matching estimators.

Matching estimator	Performance criteria		
	Balancing test⁣^∗^	Pseudo-*R*^2^	Matched sample size
Nearest neighbor			
1. Neighbor	8	0.176	208
2. Neighbor	8	0.134	208
3. Neighbor	9	0.108	208
4. Neighbor	10	0.110	208
5. Neighbor	9	0.095	208
Radius caliper			
0.01	10	0.029	208
0.1	8	0.092	208
0.25	10	0.059	136
0.5	6	0.101	136
Kernel matching			
With no band width	10	0.089	124
Band width of 0.1	9	0.093	129
Band width of 0.25	10	0.063	136
Band width of 0.5	9	0.066	136

*Note:* Source: own computational result (2018).

⁣^∗^The number of explanatory variables where there are no matched groups of adopter households and nonadopter households with statistically significant mean differences.

**Table 8:** Chi-square test for the joint significance of variables.

Sample	Ps *R*^2^	LR chi2	*p* > chi2
Unmatched	0.323	106.63	0.001
Matched	0.029	2.90	0.992

*Note:* Source: own estimation result based on survey (2018).

**Table 9:** Average treatment effects on the treated (ATT) for energy expenditure.

Variable	Treated	Controls	Difference	S.E.	*T*-stat
Energy expenditure unmatched	1525.59	2441.21	−915.62	85.29	−10.75
Energy expenditure ATT	1935	2395.74	−460.74	169.70	−2.72

*Note:* Source: own estimation result based on survey (2018).

## Data Availability

The data that support the findings of this study are available from the corresponding author upon reasonable request.

## Endnotes

^1^Kebele is an equivalent with lowest administrative units.
